# Protein microarray-based profiling of salivary IgA antibodies against human proteins in COVID-19 patients with depressive symptoms

**DOI:** 10.1038/s41598-026-45180-2

**Published:** 2026-03-23

**Authors:** Yuichi Hikichi, Kohei Kunieda

**Affiliations:** 1 Tuning Fork Bio, Inc, 1 Broadway, Cambridge, MA 02142 USA; 2 MITSUI LINK-Lab, Tuning Fork Bio Japan, SHINKIBA 2, Room 409, 1-17-8 Shinkiba, Koto-ku, Tokyo 136-0082 Japan

**Keywords:** Biomarkers, Diseases, Immunology, Medical research, Neurology, Neuroscience

## Abstract

To understand the pathophysiology and etiology of COVID-19 patients with depressive symptoms, we investigated the presence of autoantibodies associated with neurological sequelae using salivary IgA antibody profiling in COVID-19 patients with depressive symptoms. We used a human protein microarray system to comprehensively measure IgA antibodies against human proteins in the saliva of three groups of participants: those who developed moderate or severe depressive symptoms 2–14 months after SARS-CoV-2 infection (the ‘COVID-19 group’), those with no infection history who showed no depressive symptoms or only mild depressive symptoms (the ‘healthy control group’), and those with no infection history who were diagnosed with depression. As a result, 65 types of IgA antibodies recognizing human proteins were identified, all of which were present only in the COVID-19 group and not in the healthy control group. Several antigens recognized by these antibodies are associated with depressive symptoms. These findings suggest that distinctive salivary IgA antibody profiles may be associated with depressive symptoms following SARS-CoV-2 infection and could serve as candidate biomarkers for further investigation.

**Supplementary Information:**

The online version contains supplementary material available at 10.1038/s41598-026-45180-2.

## Introduction

The coronavirus disease 2019 (COVID-19) pandemic caused by severe acute respiratory syndrome coronavirus 2 (SARS-CoV-2) was declared by the World Health Organization on March 11, 2020. As the number of infected individuals has increased, reports have emerged of severe multiorgan dysfunction with serious symptoms occurring after SARS-CoV-2 infection, referred to as Long COVID (sometimes referred to as ‘post-acute sequelae of COVID-19 [PASC]’). Long COVID affects at least 10% of infected individuals, with more than 200 reported symptoms impacting multiple organ systems^1^. Neurological and cognitive symptoms include cognitive impairment (‘brain fog’), fatigue, memory loss, sensory hypersensitivity (sensitivity to light or sound), and autonomic dysfunction (e.g., postural orthostatic tachycardia syndrome [POTS]). These symptoms are similar to those of depression. Epidemiological analyses have shown an association between increases in depression and anxiety disorders and Long COVID^2–5^. The precise mechanism linking SARS-CoV-2 infection to the onset of these symptoms remains unknown.

Long COVID is now understood as a heterogeneous and multifactorial condition rather than a single disease entity. Proposed mechanisms include persistent immune dysregulation, chronic inflammation, viral persistence, autonomic dysfunction, and the generation of autoantibodies following SARS-CoV-2 infection^1^. Among these, immune-mediated pathways have been extensively investigated, and several studies have reported elevated autoantibody responses in both acute COVID-19 and Long COVID. One possible cause of its onset is that autoantibodies produced after SARS-CoV-2 infection may trigger an autoimmune reaction through immune system priming via molecular mimicry.

In parallel, Long COVID remains difficult to diagnose because symptoms are subjective, variable, and overlap with psychiatric conditions such as depression, underscoring the need for objective and minimally invasive biomarkers^6,7^. Saliva is an attractive biofluid for this purpose due to its noninvasive collection, and IgA is the predominant immunoglobulin class in saliva, reflecting mucosal immune responses. These considerations motivated our exploratory profiling of salivary IgA antibodies in individuals with post-COVID depressive symptoms.

Depression is a clinically heterogeneous disorder encompassing diverse symptom profiles and underlying etiologies. Notably, several neuropsychiatric symptoms frequently reported in Long COVID—including fatigue, cognitive impairment (“brain fog”), sleep disturbances, and mood changes—overlap phenomenologically with symptoms assessed in major depressive disorder^8–10^. However, the presence of depressive symptoms in Long COVID does not necessarily indicate a diagnosis of major depressive disorder as defined by the Diagnostic and Statistical Manual of Mental Disorders (DSM) criteria, and these symptom constellations may reflect distinct or partially overlapping biological processes.

In patients with SARS-CoV-2 infection, autoantibody reactivity is significantly increased compared with that in non-infected patients, and autoantibodies against immune regulatory proteins (cytokines, chemokines, complement components, cell surface proteins, etc.) are detected at a greater frequency. These autoantibodies have been reported to increase disease severity^11^. Multiple studies have reported elevated autoantibody levels in patients with Long COVID patients^12^. These reports include autoantibodies against ACE2 (the entry receptor for SARS-CoV-2)^13^, β2 adrenergic receptors, muscarinic M2 receptors, angiotensin II AT1 receptors, and angiotensin I-7 MAS receptors^14^. However, no clear association has been demonstrated between Long COVID and the presence of autoantibodies. Furthermore, to our knowledge, no study has demonstrated an association between depressive symptoms and autoantibodies in patients with Long COVID. Considering the noninvasive nature of saliva collection, ease of sampling, and minimal patient burden, we focused on IgA antibodies in saliva. Using human protein microarray technology^15,16^ capable of detecting antibody binding to over 15,000 human proteins in a single assay, we investigated the presence of antibodies associated with depressive symptoms by profiling IgA antibodies in the saliva of patients diagnosed with COVID-19 who exhibited depressive symptoms. To clarify group characteristics upfront and to mitigate concerns about reverse causation, none of the participants in the COVID‑19 group had a prior clinical diagnosis of depression before SARS‑CoV‑2 infection. In this study, we use the term “IgA autoantibody” to describe salivary IgA antibody against human proteins detected by protein microarray analysis. Although such reactivities may include actual autoantibodies, the present study does not establish antigen specificity, affinity, or pathogenic significance.

## Results

### Characteristics of participants

The study cohort consisted of three groups: a COVID-19 group (*n* = 8) comprising participants with a history of SARS-CoV-2 infection and depressive symptoms, a healthy control group (*n* = 8) without infection history or depressive symptoms, and a depression group (*n* = 9) with a physician-confirmed diagnosis of depression and no history of SARS-CoV-2 infection. All available samples from each group were included in the primary analyses unless otherwise specified.

The analyses were designed to identify IgA antibody reactivity patterns that were characteristic of the COVID-19 group with depressive symptoms, in comparison with two comparator groups: healthy controls and individuals with depression without a history of SARS-CoV-2 infection. The healthy control group served as a baseline reference, while the depression group was included to distinguish COVID-19–associated immune profiles from depressive symptoms unrelated to COVID-19.

In the context of patients infected with SARS-CoV-2, saliva samples were collected from eight individuals who experienced depressive symptoms and exhibited a moderate or high risk of depression (12 points or higher) based on the PHQ-9 (Patient Health Questionnaire-9), a self-administered screening tool for depression (the ‘COVID-19 group’). COVID-19 symptoms in the group were diagnosed as mild to moderate. All participants, except one, exhibited symptoms of Long COVID, including taste disorders, hair loss, and fatigue (Supplementary Table 1). These participants were not diagnosed with depression. Saliva samples were collected from eight individuals who had no history of SARS-CoV-2 infection and a moderate risk or below (11 points or lower) on the PHQ-9 questionnaire (the ‘healthy control group’). Furthermore, saliva was collected from nine participants diagnosed with depression (the ‘depression group’) to compare depressive symptoms. Table 1 presents the age, sex, and time elapsed between infection and the PHQ-9 score for the participants in this study.

**Table 1:** Characteristics of participants.

	COVID-19	Healthy control	Depression
Number of subjects (male/female)	8 (2/6)	8 (5/3)	9 (5/4)
Age (years)	44.9 ± 6.6	46.4 ± 14.1	40.2 ± 10.5
Period from infection to sample collection (average)	2–14 months (8 months)	–	–
PHQ-9 score (average)	17.3 ± 4.8	5.1 ± 4.8	17.3 ± 8.4

### Overview of autoantibody profiling

Autoantibody profiling was performed by examining 25 saliva samples from the COVID-19, healthy control, and depression groups for salivary IgA binding to approximately 15,000 different human proteins using human protein microarray analysis (Supplementary Table 2). Candidate antigens were selected based on relative signal elevation in the COVID-19 group compared with the healthy control group, using predefined threshold-based criteria to highlight prominent IgA reactivity rather than to test for statistical significance. These criteria were applied uniformly across all samples.

In the healthy control group, no samples showed high signals, whereas in the COVID‑19 group, at least one sample showed an antigen signal ≥ 1.5 standard deviations above the group mean, identifying it as a candidate antigen for further evaluation. Furthermore, the lower limit of the COVID-19 group’s measurements and the upper limit of the healthy control group’s measurements were set, and analysis was performed under these four conditions. Because IgA signal intensities extended from values above approximately 600 to below approximately 25, we divided this continuous range into four partially overlapping intensity intervals, each defined by a two‑fold increment in both the COVID‑19 group and the healthy‑control thresholds. For each interval, the lower and upper limits were chosen to maintain at least a three‑fold separation between the two groups, allowing candidate antigens to be assessed across distinct signal ranges while preserving the robustness of the selection criteria. The results of the analysis are presented in Table 2. Under Condition 1 (COVID-19 group measurement ≥ 75, healthy control group measurement ≤ 25), three antigens were identified; under Condition 2 (COVID-19 group measurement ≥ 150, healthy control group measurement ≤ 50), 20 antigens were identified; under Condition 3 (COVID-19 group measurement ≥ 300, healthy control group measurement ≤ 100), 43 antigens were identified; and under Condition 4 (COVID-19 group measurement ≥ 600, healthy control group measurement ≤ 200), 24 antigens were identified. Notably, some proteins were represented by multiple spots on the protein microarray; in such cases, duplicate hits were counted as a single antigen. For example, under Condition 2, although 22 spots were positive, they corresponded to 20 unique antigens.

**Table 2:** Antigens (gene name) against IgA detected only in the COVID-19 group.

Condition 1	Condition 2	Condition 3	Condition 4
ANAPC15	C19orf44	ADA	ADA
C19orf44	C5orf15	ALKBH1	AGPAT4
FAM219A	CDC42EP4	ALS2CR12	ALS2CR12
	CDR2	ANKRD1	ATP5J
	CHMP2B	ATP5J	C8orf59
	CHMP7	ATPAF1	CBX8
	CREBL2	BEX5	CHMP2B
	GABPB2	BOLA3	CHMP7
	GOT1	C8orf59	FZR1
	ISL1	CA1	HLF
	LIMA1	CBX8	JDP2
	MTUS1	CCDC121	MAGEB16
	NCBP3	CCDC43	MAP6D1
	NEFH	CDC42EP4	MICU3
	PIP5K1C	CETN2	NDUFAF2
	PPP1R17	CFAP58	PDF
	SLCO4C1	CHMP2B	SH3BGRL
	SOX10	CHMP7	TMEM35B
	SPAG7	COX19	TRPM1
	TMX1	CREBL2	TSGA13
		EIF3F	UBL7
		FAM149B1	ZMIZ1
		GOT1	ZNF330
		INSL4	ZSWIM7
		INTS12	
		JDP2	
		LIMA1	
		MAGEB16	
		NCBP3	
		NDUFAF2	
		PDF	
		PFDN5	
		PIP5K1C	
		POLR3D	
		PPP1R17	
		RPL9	
		SS18	
		TMEM35B	
		VAV3	
		ZMIZ1	
		ZNF330	
		ZNF84	
		ZSWIM7	

**Fig. 1:**
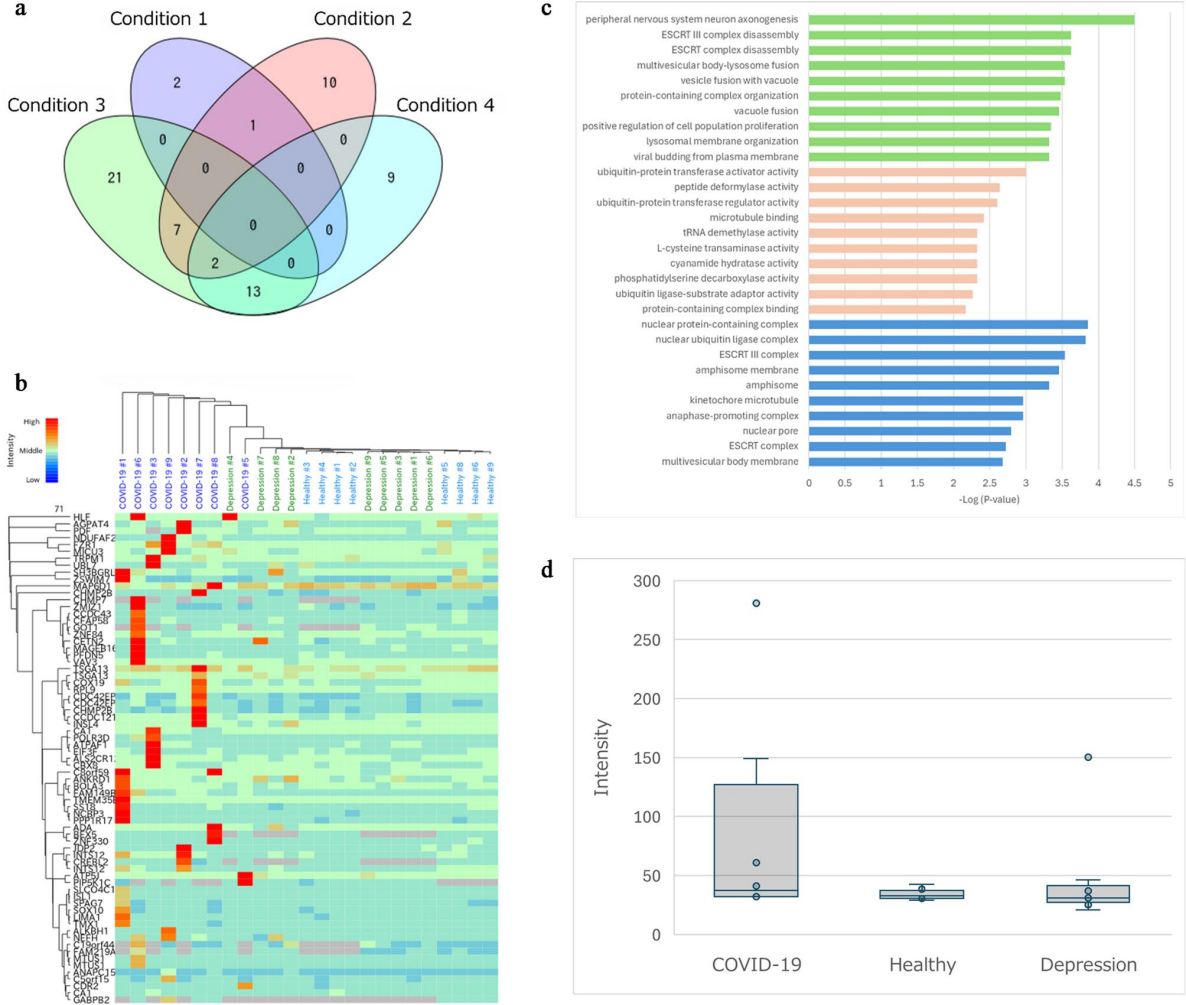
Antigen profile of IgA autoantibodies identified in the COVID-19 group. (**a**) Venn diagram of antigens identified under the four analytical conditions. (**b**) Cluster analysis using data from 71 antigen data points (corresponding to 65 unique antigens). (**c**) GO analysis of autoantibodies specific to the COVID-19 group. Green, pink, and blue indicate cellular components, molecular functions, and biological processes, respectively. (**d**) Box-and-whisker plot of anti-NEFH IgA levels in each group. The center line represents the median, the box indicates the interquartile range (IQR), and the whiskers denote the minimum and maximum values excluding outliers, as defined by the following criteria. Data points plotted outside the whiskers correspond to outliers, defined as values outside the range of Q1–1.5×IQR to Q3 + 1.5×IQR. Group sizes: COVID-19, *n* = 8; healthy controls, *n* = 8; depression, *n* = 9. All images in Fig. 1 were taken by the authors and are published under CC BY 4.0.

After excluding duplicate antigens identified under the four analysis conditions, 65 distinct antigens were identified (Fig. 1a). None of the antigens were detected across all four analytical conditions, whereas CHMP2B and CHMP7 were detected in three of the four analyses. Twenty-one antigens were detected under two conditions: ADA, ALS2CR12, ATP5J, C19orf44, C8orf59, CBX8, CDC42EP4, CREBL2, GOT1, JDP2, LIMA1, MAGEB16, NCBP3, NDUFAF2, PDF, PIP5K1C, PPP1R17, TMEM35B, ZMIZ1, ZNF330, and ZSWIM7. The depression group was analyzed in parallel to assess whether observed IgA profiles were specific to the COVID-19 group or were shared with depression in the absence of SARS-CoV-2 infection. We additionally examined whether these proteins were detected in the depression group; however, the selection of the ten proteins in Table 3 was based solely on prior literature.

**Table 3:** Antigens previously reported to be associated with neurological or depressive symptoms in the literature.

Gene	Type	Function	Symptoms	References
CHMP2B	Membrane	Endolysosomal transport, viral budding (including SARS-CoV-2)	Depression	Chassefeyre et al.^17^ Keeley et al.^18^
CHMP7	Membrane	Nuclear pore complex repair, Neural progenitor cell surveillance, Viral replication control	Cognitive impairment	Keeley et al.^18^ Coyne et al.^19^
NEFH	Cytoskeleton	Neurofilament H, Axonal Stability	Ataxia	Khalil et al.^20^
CDR2	Cytoplasm	Intracellular signal transduction, synaptic plasticity, and regulation of cellular homeostasis	Emotional disorder	Martínez Lozada et al.^21^
TRPM1	Membrane channel	Visual communication	Visual impairment	Hung et al.^22^
CNGA2	Membrane channel	Olfactory transmission	Olfactory dysfunction	Biel et al.^23^
CLIC5	Membrane	In the hair cells of the inner ear, maintaining the stability of stereocilia and participating in auditory transmission	Dizziness	Ott et al.^24^
CETN2	Cilium	Maintenance of the structure of olfactory cilia	Olfactory dysfunction	Ying et al.^25^
CFAP58	Cilium	Formation and stability of motile cilia	Olfactory dysfunction	He et al.^26^
FAM149B1	Cilium	Formation and stability of primary cilia	Ataxia, Olfactory dysfunction	Shaheen et al.^27^

Hierarchical clustering analysis using data from 71 antigen data points (corresponding to 65 unique antigens) showed that samples from the COVID-19 group generally clustered together and were distinct from those of the healthy control and depression groups, despite some variability in inter-sample distances (Fig. 1b).

### IgA autoantibodies specific for COVID-19 patients with depressive symptoms

We performed gene ontology classification and enrichment analysis using 65 antigen proteins identified against saliva-derived IgA in the COVID-19 group with depressive symptoms. These proteins were enriched in peripheral nervous system neuron axonogenesis, ESCRT complex disassembly, and ESCRT III complex disassembly (Fig. 1c). Analysis of biological processes revealed gene ontology terms related to ubiquitin-protein transferase activator activity. For molecular function, 11 antigenic genes were classified as nuclear protein-containing complexes. The details of the GO analysis are presented in Supplementary Table 3. Based on these results, we were able to identify autoantibodies against NEFH, a major cytoskeletal protein supporting the structural framework of nerve cells included in the GO term “peripheral nervous system neuron axonogenesis.” This autoantibody was detected in three samples from the COVID-19 group, was not detected in the healthy group, and was detected in one sample from the depression group (Fig. 1d).

Among the 65 proteins identified as antigens for autoantibodies, 10 proteins considered relevant to depressive symptoms were selected based on prior literature, as summarized in Table 3. The functional suppression of these proteins was correlated with emotional disturbances, dizziness, and cognitive impairment, which are accompanied by depressive symptoms. Furthermore, it also contains the proteins CNGA2, CETN2, CFAP58, and FAM149B1, which are associated with olfactory dysfunction, a typical symptom of SARS-CoV-2 infection. Autoantibodies against these proteins may cause depressive symptoms in Long COVID patients.

These analyses were intended to describe relative differences in IgA antibody profiles across groups and to identify candidate features associated with post-COVID depressive symptoms, without implying causality or diagnostic validity.

## Discussion

The present study was designed as an exploratory investigation within the broader immunological framework of Long COVID. In light of the diagnostic complexity of Long COVID and the lack of established biomarkers for neuropsychiatric symptoms, we focused on salivary IgA antibody profiles as a noninvasive immune signature. Our findings should therefore be interpreted primarily as hypothesis-generating and as identifying candidate biomarkers, rather than as evidence of causality or direct pathogenic mechanisms.

The WHO definition of Long COVID refers to symptoms that typically begin within 3 months of COVID-19 onset, persist for at least 2 months, and cannot be explained by other diagnoses^28^. Symptoms vary and may include fatigue, shortness of breath, muscle and joint pain, and sleep disorders. The participants in this study included individuals whose symptoms had persisted for less than 2 months, but they exhibited symptoms of Long COVID, particularly depressive symptoms. By analyzing autoantibodies in samples from these patients, we believe that we can gain insight into one aspect of the cause of depressive symptoms in patients with Long COVID. The current definition and diagnostic criteria for Long COVID are still evolving, and there is no universally accepted biomarker or single pathophysiological mechanism. Long COVID is increasingly recognized as a heterogeneous condition with multiple potential etiologies rather than a uniform disease entity. In this context, our study adopted an exploratory approach: rather than focusing solely on antigens showing statistically significant differences between groups, we prioritized autoantibodies that exhibited markedly elevated levels (outlier-like patterns) in individuals within the COVID-19 group. This strategy is reasonable, given the likely heterogeneity of Long COVID and the possibility that distinct subgroups may be driven by different immunological mechanisms.

We aim to validate these candidate autoantibody markers via multiplex assays in larger, independent cohorts. Such studies will allow us to assess the reproducibility, clinical relevance, and potential utility of these markers as diagnostic or prognostic indicators of Long COVID with neuropsychiatric symptoms. Furthermore, one purpose of analyzing IgA antibodies in saliva via noninvasive saliva samples collected from participants is to facilitate future diagnostic applications and identify factors associated with depressive symptoms. Accordingly, depressive symptoms observed in the COVID-19 group should be interpreted as symptom‑based manifestations rather than as equivalent to a formal psychiatric diagnosis, consistent with the exploratory and biomarker‑oriented scope of this study.

Inflammation represents a shared biological feature of both COVID-19 and major depressive disorder and has been implicated in neuropsychiatric manifestations in each condition. Systemic and persistent inflammatory responses following SARS-CoV-2 infection may influence neural function through indirect mechanisms, including immune–neural interactions and chronic immune activation. In this context, the salivary IgA reactivity patterns observed in the present study may reflect an inflammation-associated immune state rather than disease-specific pathogenic effects.

Analysis of IgA present in the saliva of the COVID-19 group revealed 65 types of IgA autoantibodies. Among these, several proteins were previously reported to be associated with depressive symptoms (Table 3). We focused on NEFH, CHMP2B, and CHMP7. NEFH (neurofilament heavy chain) is one of the major cytoskeletal proteins supporting the axonal structure of nerve cells and is a key factor in axonal structural stabilization, the regulation of nerve conduction velocity, and nerve maturation^15,29^. Abnormalities in NEFH levels are also associated with diseases. Additionally, neurofilament light chain (NfL), another constituent protein of neurofilaments, has been reported to be a blood factor that significantly increases in patients with Long COVID-related neurological impairment^30,31^. Therefore, in some patients with Long COVID, neurofilament structural proteins may leak into the bloodstream, potentially triggering the production of antibodies against these proteins. However, the present study does not provide evidence that such antibodies functionally inhibit neurofilament proteins or directly induce depressive symptoms.

Several antigenic proteins identified in this study have been previously implicated in neurological or neuropsychiatric processes^32,33^. However, the present findings do not provide direct evidence that IgA antibodies against these proteins exert functional inhibition or causally induce depressive symptoms. Rather, the observed IgA reactivities should be interpreted as immune correlates that may reflect underlying biological processes associated with post-COVID- depressive symptoms. In particular, for intracellular proteins such as CHMP2B, and CHMP7, antibody binding is more likely to represent a biomarker of immune activation or tissue perturbation rather than a direct pathogenic mechanism.

CHMP2B and CHMP7 are proteins involved in the endosomal-sorted complex required for transport (ESCRT) complex. The ESCRT complex is essential for the budding of enveloped viruses and is involved in the particle formation and release of SARS-CoV-2^17–19^. Furthermore, ESCRT complex dysfunction has been suggested to potentially induce neurodegeneration and inflammation. Although autoantibodies against these proteins were identified in our study, they are unlikely to directly inhibit viral budding because these antigens are intracellular and generally inaccessible to antibodies. However, ESCRT dysfunction has been implicated in neurodegeneration and may influence viral particle clearance. Persistent SARS-CoV-2 RNA or viral components have been reported in the tissues of patients with Long COVID, suggesting that incomplete viral egress could contribute to chronic immune activation. Therefore, autoantibodies targeting ESCRT-related proteins may reflect or exacerbate cellular stress pathways that indirectly affect viral clearance and promote prolonged inflammatory states.

Therefore, autoantibodies against CHMP2B and CHMP7 are likely elevated in patients with Long COVID who exhibit depressive symptoms.

A previously published study used a protein microarray to identify IgG autoantibodies in the blood of patients with Long COVID with neurological symptoms^34^. This study identified 42 different autoantibodies. However, only the antigenic protein for SOX10 matched the IgA autoantibodies identified in our study. The reason for this discrepancy is unclear but may be due to differences in the antibody classes examined or variations in the protein microarray used for detection.

This study identified ten types of autoantibodies against proteins associated with depression symptoms. Furthermore, autoantibodies against NEFH, CHMP2B, and CHMP7, which are proteins directly associated with depressive symptoms, were obtained. It is necessary to increase the number of participants and conduct a more detailed evaluation of the associations between these autoantibodies and depressive symptoms. These autoantibody profiles may serve as candidate biomarkers for future studies aimed at understanding and stratifying post-COVID depressive symptomatology.

This study has several limitations. First, the cohort size was modest and clinically heterogeneous. Second, current medication and supplement use were collected by self-report and were not adjusted for in the primary analyses; psychotropic or other medications could potentially influence salivary IgA profiles and represent an uncontrolled source of variability. Third, saliva and questionnaire data were obtained at a single time point, and the interval between infection and sampling varied among participants in the COVID-19 group, which may have introduced temporal effects on immune readouts. These factors should be considered when interpreting the findings, and future studies with larger, longitudinal cohorts and prespecified medication stratification are warranted-. In addition, this study focused on profiling IgA reactivity patterns using a protein microarray platform and did not quantify total salivary IgA levels or validate individual antigen–antibody interactions by orthogonal assays such as ELISA. Therefore, the present results do not distinguish between increased IgA abundance and changes in antigen-specific IgA recognition. -Future studies incorporating quantitative measurements and independent validation assays will be necessary to further characterize the biological significance of these findings.

## Methods

### Study design

In March 2022, Japanese individuals aged ≥ 20 years participated in this study. Approximately 8–9 participants were enrolled in each of the three groups: the COVID-19, healthy control, and depression groups. The participants completed self-administered questionnaires regarding their SARS-CoV-2 infection status, COVID-19 symptoms, and Long COVID symptoms. The self-administered questionnaire was designed by the authors and does not incorporate third party copyrighted items.

The depression group comprised participants who were diagnosed with depression by a physician. The healthy control and depression groups were used for comparisons with the COVID-19 group; however, comparisons between the two comparator groups themselves were not conducted in this report.

Depressive symptoms were assessed using the Patient Health Questionnaire-9 (PHQ-9), a validated self-report instrument for symptom severity screening, which is distributed by Pfizer without copyright restriction; no permission is required for its reproduction.

PHQ-9 scores were used in this study to quantify the presence and severity of depressive symptoms rather than to establish a clinical diagnosis of major depressive disorder.

PHQ-9 questionnaires were collected contemporaneously with saliva sampling as part of the same assessment session for all participants.

The participants completed the PHQ-9 questionnaire and sent self-collected saliva samples, which were obtained using a SimplOFy saliva collection kit (Oasis Diagnostics, WA, USA), to the institution for protein microarray analysis. These saliva samples were used for IgA autoantibody profiling using a human protein microarray.

Eligibility criteria for the present analysis were as follows. All participants were aged ≥ 20 years and provided written informed consent. Individuals in the COVID-19 group reported a history of SARS-CoV-2 infection and endorsed depressive symptoms; those with a prior clinical diagnosis of depression before SARS-CoV-2 infection were not eligible for the COVID-19 group. Healthy controls reported no history of SARS-CoV-2 infection and no physician-diagnosed depression, and the depression group comprised participants with a physician-confirmed diagnosis of depression and no history of SARS-CoV-2 infection. Basic demographics (age and sex), infection history and post-COVID symptomatology, and PHQ-9 scores were collected using structured self-report questionnaires.

Medication and supplement use were ascertained via structured items that requested the names and dosages of all current prescription and over-the-counter drugs and the names and start dates of supplements or health foods. These data were recorded for all groups based on participant self-report. Given the small sample size and heterogeneity of the cohorts, medication status was not modeled as a covariate in the primary analysis; instead, its potential impact is addressed in the Limitations.

Saliva and questionnaire data were collected at a single time point per participant. In the COVID-19 group, the interval between SARS-CoV-2 infection and sample collection varied across individuals, reflecting real-world recruitment.

The study was approved by the Japan Conference of the Clinical Research Ethics Committee on February 18, 2022. This study conformed to the Declaration of Helsinki and the Code of Ethics of the World Medical Association.

Written informed consent was obtained from all participants prior to sample collection and questionnaire completion.

### Human protein microarray

Saliva (target volume: 1.0 mL per participant) was collected at home using a standardized collection kit in the early morning before food intake or tooth brushing, according to the written instructions provided to participants. Samples were subsequently shipped to the testing facility under the study’s anonymization procedure. Salivary IgA antibody screening was performed using human protein microarrays contracted by the Fukushima Translational Research Project in Fukushima, Japan^10,11^. The protein microarray contained 16,680 full-length human proteins, encompassing 15,406 genes, including 1190 malignancy-associated mutants and fusion proteins. To evaluate salivary autoantibodies, we excluded malignancy-associated mutants and fusion proteins and used 15,272 full-length human proteins. Human protein microarray analysis was performed according to the manufacturer’s protocol. In brief, the microarrays were incubated with diluted saliva (2 µL of saliva diluted 1000-fold) and Goat Reference Antibody Mixture I (Fukushima Protein Factory, Inc., Fukushima, Japan) after blocking, and stained with Alexa Fluor 647-conjugated anti-human IgA and Cy3-conjugated anti-goat IgG antibodies, respectively. After staining, the microarrays were scanned using a GenePix 4000 B scanner (Axon Instruments), and those not incubated with saliva were used as negative controls. To compare the microarray data, the fluorescence intensity was normalized using the following method: all fluorescence intensities were converted to log_2_ values, with negative values treated as missing. The background (Cy3) value was subtracted from the autoantibody detection (Alexa Fluor 647) value. Within each sample, the top and bottom 8% of values were trimmed to reduce the influence of extreme outliers. The mean was subtracted from each value. For each sample in each measurement, the negative control value was subtracted. Finally, quantile normalization was performed. This normalization method, named Tailored Expression Adjustment for Autoantibody in Liquid (TEAL), was specifically designed to minimize the technical variability and background noise inherent to protein microarrays and to improve comparability across heterogeneous samples.

### Data analysis

To identify autoantibodies specific to the COVID-19 group, we selected antigens for which at least one case in the COVID-19 group showed a value at least 1.5 standard deviations above the group mean. Furthermore, a positive antigen was defined as one with a value at least three times higher than the maximum measurement in the healthy control group.

Hierarchical clustering analysis was performed using IgA measurements for 71 antigen data points (corresponding to 65 unique antigens). The dissimilarity between samples was calculated using the Euclidean distance, and cluster analysis was performed using Ward’s minimum variance method, which minimizes the total within-cluster variance at each step of the agglomeration process.

Gene Ontology classification and enrichment analyses were performed using the software (Tuning-Base™) we developed in-house. Fisher’s exact test was used to measure gene enrichment in the annotation terms. The top 10 Gene Ontology classifications, sorted by p value from highest to lowest, are shown.

The box-and-whisker plot, in which the center line represents the median, the box indicates the interquartile range (IQR), and the whiskers denote the minimum and maximum values, was created using Microsoft Excel (Microsoft Corporation, WA, USA). Data values outside the range defined by the first quartile (Q1) minus 1.5×IQR and the third quartile (Q3) plus 1.5×IQR were treated as outliers.

### Use of artificial intelligence tools

Hierarchical clustering was performed using an unsupervised machine learning algorithm (Ward’s method with Euclidean distance). While this is a standard statistical approach, it is considered part of AI-based data analysis techniques.

GPT‑5 (OpenAI, SF, USA) was also employed to support language refinement and structural review of the manuscript draft. No content was generated without author verification, and all scientific interpretations and conclusions were made by the authors.

## Supplementary Information

Below is the link to the electronic supplementary material.

Supplementary Material 1

Supplementary Material 2

Supplementary Material 3

## Data Availability

The data that support the findings of this study are available from Tuning Fork Bio, Inc. However, restrictions apply to the availability of these data, which were used under license for the current study and are not publicly available. Data are, however, available from the authors upon reasonable request and with permission from Tuning Fork Bio, Inc.
